# Multiple adenomas of the thyroid gland in an African green monkey (*Chlorocebus aethiops*)

**DOI:** 10.5194/pb-10-1-2023

**Published:** 2023-05-12

**Authors:** Roland Plesker, Kernt Köhler

**Affiliations:** 1 Zentrale Tierhaltung, Paul-Ehrlich-Institut, Langen, Germany​​​​​​​; 2 Institut für Veterinär-Pathologie, Justus-Liebig-Universität, Gießen, Germany

## Abstract

Two cystadenomas and one solid adenoma of the thyroid gland in a 27-year-old female African green monkey (*Chlorocebus aethiops*) are described here. Histologically, the solid adenoma was classified as a well-defined solid follicular adenoma of microfollicular type. The solid adenoma was positive for thyroglobulin in immunohistochemistry staining, whereas the cystadenomas stained positive for both thyroglobulin and calcitonin. No evidence of excess hormone production related to the tumor presence was detected.

## 1 Introduction and literature overview

Neoplasia of the endocrine system in nonhuman primates is relatively uncommon. When it does occur, the majority of reported endocrine tumors tend to be benign and nonfunctional (Miller, 2012). Thyroid gland neoplasms are particularly rare (Anderson and Capen, 1978). Tumors of the thyroid gland have been reported mainly in macaques and consisted of both adenomas and adenocarcinomas (Beniashvili, 1989). However, the majority of tumors of the thyroid gland are reported to be adenomas (Suckow et al., 2021; Simmons, 2016; Scott, 1992).

Thyroid gland adenomas and cystadenomas have been described in several species of prosimians, most notably in the Sanford's brown lemur (*Eulemur sanfordi*) and in the crowned lemur (*Eulemur coronatus*) (Remick et al., 2009).

In nonhuman primates, we counted a total of 49 reports of thyroid gland adenomas and 17 cases of thyroid gland carcinomas in the literature.

In rhesus macaques (*Macaca mulatta*), Colgin et al. (2016) described a thyroid gland parafollicular cell adenoma, while Simmons (2016) and Simmons and Mattison (2011) observed a total of nine thyroid gland adenomas and two C-cell carcinomas in rhesus macaques. Uno (1997) reported a papillary adenocarcinoma of the thyroid gland when describing age-related pathology in captive rhesus macaques. Amongst other species, Lowenstine (1986) named eight thyroid gland adenomas and one metastatic carcinoma in macaques in her survey of neoplasms and proliferative disorders in nonhuman primates. McClure (1975) first reported a well-differentiated adenocarcinoma of the thyroid gland in an 18-year-old female rhesus macaque and later, in McClure (1980), multiple adenomas in another rhesus macaque. Both macaques had a history of radiation exposure. Kraft (1971) found a thyroid gland carcinoma in a rhesus macaque. Yakovleva (1964) observed a thyroid gland adenoma in a 10-year-old male rhesus macaque after irradiation.

In cynomolgus macaques (*Macaca fascicularis*), cases of both C-cell carcinoma and thyroid gland follicular adenoma have been reported (Kaspareit et al., 2007).

When investigating the prevalence of endocrine neoplasia-like syndrome in baboons (*Papio* spp.) at the Southwest National Primate Research Center (SNPRC) in Texas, Confer et al. (2018) reported four adenomas and two carcinomas of the thyroid gland. When evaluating 434 endocrine-related diagnoses from 4619 necropsies of baboons at the SNPRC, Guardado-Mendoza et al. (2009) listed 11 thyroid gland adenomas and 8 carcinomas of the thyroid gland. Weber and Greeff (1973) observed four microscopic adenomas of the thyroid gland in Chacma baboons (*Papio ursinus*). Fox (1936)​​​​​​​ reported a thyroid gland adenoma in parallel with a fibroma in the omentum, in a 30-year-old male baboon (*Papio porcarius*).

Concerning New World monkeys, Kawasako et al. (2014) described a thyroid gland follicular adenoma in a 10-year-old male common marmoset (*Callithrix jacchus*). David et al. (2009) reported a case of thyroid gland adenoma in a female common marmoset in their survey of pathology of common marmosets and tamarins. Miller et al. (2009) described two cases of concurrent thyroid gland cystadenomas in a cohort of cotton-top tamarins (Saguinus oedipus) with pheochromocytomas. Dias et al. (1996)​​​​​​​ reported two cases of cystadenomas in black-tailed marmosets (*Mico melanurus*), while Lowenstine (1986) described a thyroid gland adenoma in a female squirrel monkey (*Saimiri sciureus*) and – amongst other endocrine tumors – a thyroid chief cell adenoma in a 14-year-old male mantled howler monkey (*Alouatta villosa*). A follicular adenoma was reported in a 7-month-old female patas monkey (*Erythrocebus patas*) (Ippen and Wildner, 1984). Finally, Williamson and Hunt (1970)​​​​​​​ observed a thyroid gland adenocarcinoma without metastasis in a mature female black-mantled tamarin (*Saguinus nigricollis*).

Herein, we report a case of a solid follicular adenoma and two cystadenomas of the thyroid gland in a 27-year-old female African green monkey (AGM).

## 2 Animal and methods

### 2.1 Animal provenance

The animal was a female, 27-year-old African green monkey (grivet; *Chlorocebus aethiops*). As previously described for other primates (Plesker and Hintereder, 2021; Plesker et al., 2020), the monkey was born at the Paul-Ehrlich-Institut in Langen, Germany, where it lived in an experimental indoor facility. It was group-housed in accordance with European and German animal welfare legislation.

### 2.2 Animal housing

The primate housing at the institution is also described, for example, in Plesker and Berger (2020) and Plesker et al. (2018). Briefly, the cage was made of steel with a size of 300 cm × 375 cm × 225 cm. Large windows allowed the monkey to watch the outside environment. Natural branches, ropes, nets, bedding, mirrors, Kong toys, puzzle feeders, Prima-Hedrons, music, and television were supplied for environmental enrichment. The diet consisted of monkey pellets ad libitum (Trio Munch^®^, Special Diet Services/Mazuri, Witham, England) in the morning and seasonal vegetables and fruits twice weekly in the afternoon. The monkey also received a mixture of nuts, mealworms, rice, popcorn, and curd.

### 2.3 Clinical history

The monkey had been experimentally infected with simian immunodeficiency virus (SIVagm) for 21 years. It had a history of bite wounds and tested positive for *Blastocystis hominis* twice during its lifetime. In the last 5 years of its life, the AGM was severely emaciated, and atrophy of the muscles was evident. Circulatory problems were recorded twice in the last 1.5 years of its life. Dramatic circulatory problems, combined with apathy and a generally poor prognosis, were ultimately the reasons for the euthanasia of the AGM with ketamine/xylacine and T 61 (Intervet Deutschland GmbH, Unterschleißheim, Germany).

The monkey lived in a long-lasting post-experimental housing phase at the Paul-Ehrlich-Institut in accordance with the German housing permission. No experimental procedures were performed for many years and the reported pathological findings were spontaneous, unexpected and accidental findings at the necropsy.

The monkey had no history of irradiation. Clinically, no signs of hyperactivity, dyspnea, cuffing, goiter, or enlargement of cervical lymph nodes were seen.

### 2.4 Necropsy

As in other cases (e.g., Plesker et al., 2020, 2018), necropsy was performed immediately after the death of the animal. Photographs were taken, and organs of interest were fixed in 4 % formaldehyde solution for 3 d before processing. Paraffin embedding of fixed tissues, preparation of 4 µ m sections, and hematoxylin and eosin (H&E) staining were performed in accordance with standard procedures.

### 2.5 Immunohistochemistry

Immunohistochemistry was performed with 4 µ m sections of the tissues fixed in 4 % buffered formalin and embedded in paraffin. After antigen retrieval and blocking, sections were incubated with polyclonal antibodies (thyroglobulin) or primary antibodies (calcitonin) (Dako-Agilent, Waldbronn, Germany). Binding was visualized either with the peroxidase–antiperoxidase method using diaminobenzidine (thyroglobulin) or with the avidin–biotin complex technique with alkaline phosphatase using Fast Red (Dako-Agilent, Waldbronn, Germany) as the chromogen (calcitonin). For the thyroglobulin detection, fixed, non-diseased tissue of AGMs and the thyroid gland of a dog served as controls. As negative control, rabbit serum or a monoclonal antibody directed against chicken lymphocytes (Hirschberger, 1987) was used. For the calcitonin detection, both a human C-cell carcinoma (positive control) and normal human thyroid gland tissue (negative control) was used to demonstrate the specificity of the antibodies.

## 3 Results

### 3.1 Necropsy

**Figure 1:**
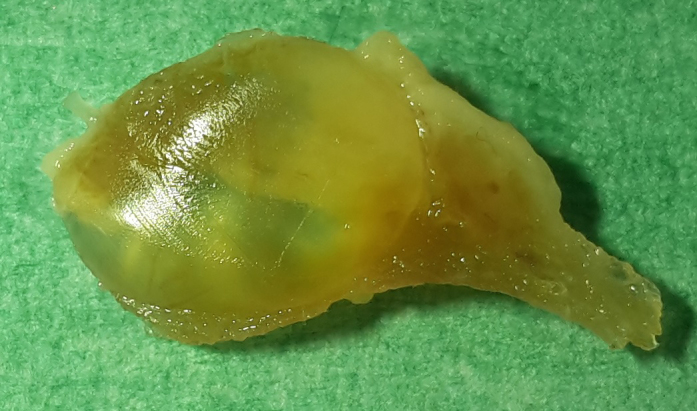
Formalin-fixed cystadenoma 10 mm in diameter of the right thyroid gland of a 27-year-old African green monkey (*Chlorocebus aethiops*).

**Figure 2:**
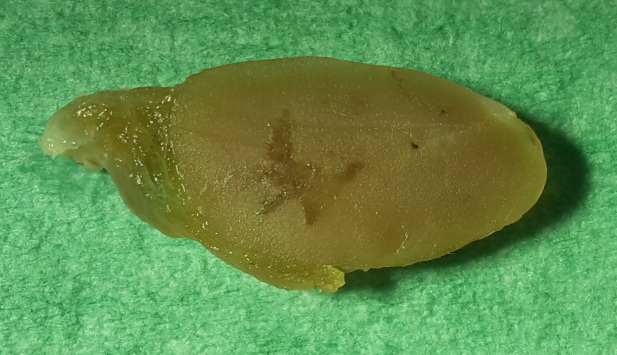
Cross section of the formalin-fixed left thyroid gland of a 27-year-old African green monkey (*Chlorocebus aethiops*): solid follicular adenoma (13 mm in length, 6.5 mm in width).

At necropsy, the monkey appeared emaciated and displayed severe general atrophy of the muscles (amyotrophia). Both thyroid glands were moderately enlarged. Macroscopically, in the right thyroid gland, two thin-walled cysts were visible with a maximum diameter of up to 10 mm (Fig. 1). In a few small sections of the cyst walls, a very small amount of adherent tumor tissue was visible. In the left thyroid gland, a tan, homogeneous, soft-elastic tumor (13 mm in diameter) with an irregular central cavity was detected (Fig. 2). In addition, the spleen was significantly enlarged by a follicular hyperplasia, and a few small cysts were seen in the ovaries. No abnormalities were detected in other organs (including the lymph nodes of the head and neck).

### 3.2 Histopathology

**Figure 3:**
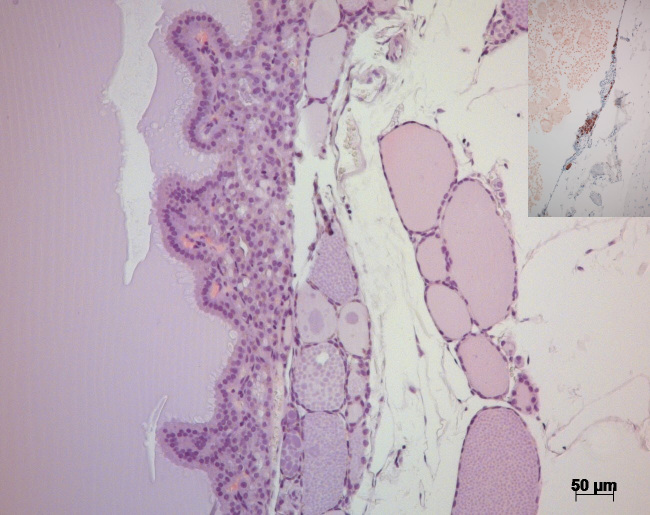
H&E stain of the thyroid gland of a 27-year-old African green monkey (*Chlorocebus aethiops*): border between a cystadenoma and residual thyroid gland tissue. Insert: immunohistochemistry – cells in the cystic wall staining positive for calcitonin.

Both cystic structures in the right thyroid gland were confirmed to be cystadenomas, with several layers of tumor cells in some sections of the walls underneath the follicular epithelium lining (Fig. 3). In the remaining thyroid tissue of the right thyroid gland, an area with hyperplastic follicular epithelium was detected, and follicles of irregular size were observed.

**Figure 4:**
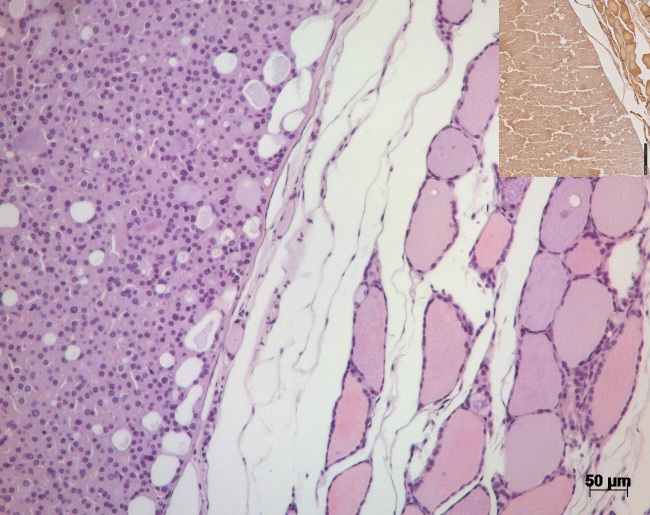
H&E stain of the thyroid gland of a 27-year-old African green monkey (*Chlorocebus aethiops*): border between a solid follicular adenoma and residual thyroid gland tissue. Insert: immunohistochemistry – solid adenoma staining positive for thyroglobulin.

In the left thyroid gland, a well-defined solid follicular adenoma with a thin fibrous connective tissue capsule was visible (Fig. 4), replacing residual follicles of the thyroid gland. The adenoma consisted of homogenous, mid-sized cells with eosinophilic cytoplasm resembling normal follicular cells of the thyroid gland. Nuclei were uniform and round. Multiple empty vacuoles (30–50 µ m in diameter) resembling incomplete and irregular follicles were distributed throughout the tumor. The central cavity of the tumor was filled with a protein-rich fluid.

In the spleen, massive follicular hyperplasia was seen, and slight atherosclerotic thickening of the media was detected in some arteries of the lungs and the heart. Additionally, some age-related changes were apparent in the kidneys (segmental sclerosis in the kidney cortex) and the liver (perivascular and parenchymal lymphatic infiltration).

### 3.3 Immunohistochemistry

Although the solid thyroid gland adenoma only stained positive for thyroglobulin, the cystadenomas were positive for thyroglobulin and, focally, for calcitonin.

## 4 Discussion

Clinically, the 27-year-old female African green monkey gave no conclusive indications of the occurrence of tumors in the thyroid gland: the monkey was not hyperactive, as could be expected in animals that produce elevated levels of thyroxine due to an endocrine active tumor of the thyroid gland (hyperthyroidism). In a retrospective investigation of stored frozen serum (obtained 1.5 years before the detection of the tumors), no elevated serum-thyroglobulin levels were detected (data not shown). The animal did not display swelling or associated volume-demanding processes in the neck, as can be seen for goiters or enlargement of cervical lymph nodes. This is in accordance with the fact that thyroid lobes affected by adenomas are usually only moderately enlarged (Rosol and Gröne, 2016). No secondary complications due to tumor-associated processes in the neck, e.g., cuffing or dyspnea, were observable. No previous thyroid-gland-associated illness had been reported in the life history of this AGM. In addition, the monkey had no history of radiation exposure/radiotherapy, which is one major risk factor for thyroid gland tumor development in humans (American Cancer Society, 2020). Since this is the first case of thyroid gland tumors in our colony, we are unable to confirm any potential heritable genetic causes which may underlie the observed tumor development, as has been reported in humans (American Cancer Society, 2020). However, this AGM was a female and this increases the risk for thyroid gland tumors, at least in humans: thyroid gland cancers are 3 times more likely to occur in women than in men (American Cancer Society, 2020). In addition, the age of the AGM might be another risk factor that potentially contributed to the tumor development in this case, since tumors in nonhuman primates tend to occur more frequently in older individuals (Lapin and Yakovleva, 2014; Beniashvili, 1989). One could argue that the emaciation and general atrophy of the muscles seen in this monkey might be an indication of an existing tumor. However, both the age of the AGM and the chronic SIVagm infection may have also contributed to these symptoms. The SIVagm infection is likely the reason for the follicular hyperplasia of the spleen in this case. In addition, this AGM showed some age-related pathological changes such as atherosclerotic thickening of the media in some arteries and sclerotic changes in the interstitium of the kidneys.

As previously mentioned in the introduction, most tumors of the thyroid gland in nonhuman primates are reported to be adenomas (Suckow et al., 2021; Simmons, 2016; Scott, 1992). In domestic animals, most thyroid gland tumors are of follicular origin (Rosol and Gröne, 2016). Follicular adenomas often develop in a thyroid gland with multinodular hyperplasia (Rosol and Gröne, 2016). Macroscopically, solid adenomas are usually sharply demarcated and either partially or completely encapsulated by a fibrous capsule of variable thickness (Rosol and Gröne, 2016). In most cases, there is only a single adenoma in a thyroid gland lobe. The thyroid gland lobe is normally only moderately enlarged and distorted, since most adenomas are relatively small. Adenomas are typically white to tan in color and appear as solid nodules that might compress adjacent thyroid follicles (Rosol and Gröne, 2016).

A different appearance is identified macroscopically in cystadenomas: they usually consist of one to two large thin-walled cavities filled with a proteinaceous fluid. The external surface is normally smooth and covered by an extensive network of blood vessels (Rosol and Gröne, 2016). These cysts usually compress residual thyroid follicles. Small masses of neoplastic tissue can remain in the wall and form rugose projections into the cyst lumen (Rosol and Gröne, 2016). These accumulations of tumor cells might even be visible macroscopically in the cyst wall.

In contrast, thyroid gland carcinomas are usually larger than adenomas, and they often contain central areas of hemorrhage and necrosis (Rosol and Gröne, 2016). Carcinomas might either invade vessels (and thereby metastasize) or invade local structures like trachea, esophagus, or surrounding muscles. Histologically, carcinomas are usually highly cellular and show more cellular pleomorphism than adenomas. Mineralization or bone formation may occur in some cases.

Histologically, solid adenomas show different growth patterns: they are either of a follicular type or of a papillary type. Adenomas derived from follicular cells that retain the ability to form follicles are considerably more common in animals than papillary adenomas (Rosol and Gröne, 2016). Each follicular adenoma has a consistent growth pattern within itself (Rosol and Gröne, 2016). The WHO classification of the endocrine system of domestic animals makes a distinction between macrofollicular and microfollicular growth patterns in solid thyroid gland adenomas (Kiupel et al., 2008). In this context, microfollicular adenomas are defined by tumor cells that arrange in miniature follicles, with either a small amount of colloid or absence of colloid (Rosol and Gröne, 2016).

In our case, the solid tumor was classified as solid follicular adenoma of microfollicular type, according to the WHO classification. The two other fluid-filled tumors were classified as cystadenomas with small sections where a narrow compact layer of tumor cells was found underneath the follicular cell lining. It might be subjective to distinguish histologically between cysts and cystadenomas (Kiupel et al., 2008).

In addition to the adenomas, a focal hyperplasia of the thyroid gland was observed, hinting that the organ might have received multiple proliferation signals inducing cell growth.

In animals other than humans, e.g., cats, this occurs in hyperthyroidism due to a thyroid gland tumor: when serum hormone levels of T4 and T3 are increased, individuals show weight loss, polydipsia and polyuria, increased defecation with an increased volume of stool, and sometimes increased physical activity (Rosol and Gröne, 2016).

Using immunohistochemistry, thyroglobulin levels in thyroid gland adenomas might vary. In humans, thyroglobulin might be decreased in thyroid gland adenomas (Valenta and Lemarchand-Beraud, 1970). In contrast, cats with follicular cell adenomas often develop hyperthyroidism, and guinea pigs might also be affected (Rosol and Gröne, 2016). In baboons, Guardado-Mendoza et al. (2009) found thyroid gland neoplastic cells which stained negative for thyroglobulin.

## 5 Conclusion

To our knowledge, this report represents the first description of thyroid gland adenomas in an African green monkey (*Chlorocebus aethiops*). Two cystadenomas as well as a solid follicular adenoma are described in a 27-year-old female. No indications of excessive hormone production due to the tumors were detected.

## Data Availability

Paraffin-embedded organ material is available via the corresponding author.
